# Epidemiology of Primary Headache and Its Associated Psychosocial Factors Amongst Undergraduate Medical Students: A Cross-Sectional Study From the Vidarbha Region

**DOI:** 10.7759/cureus.39456

**Published:** 2023-05-24

**Authors:** Sarita K Sharma, Ujwala U Ukey

**Affiliations:** 1 Community Medicine, Government Medical College and Hospital, Nagpur, IND; 2 Community Medicine, Government Medical College Nagpur, Nagpur, IND

**Keywords:** undergraduate medical, a cross-sectional study, medical students, psychosocial factors, primary headaches

## Abstract

Introduction

The present study was carried out to investigate the epidemiology of primary headaches amongst undergraduate medical students by determining the prevalence of primary headaches and their associated psychosocial factors.

Methods

A cross‑sectional study was conducted at a medical college in the Vidarbha region of India from January 2023 to February 2023 amongst 471 medical students. Diagnosis of tension-type headache (TTH) was made according to the International Classification of Headache Disorders (ICHD-3) criteria. Data were collected by interview technique using a pre-designed and pre-tested questionnaire. that consisted of socio-demographic variables and psychosocial factors. Statistical analysis was done using SPSS 24.0 (IBM Corp., Armonk, NY, USA).

Results

Prevalence of headache was 80% and was higher in females (87%) than in males (71%). TTH is the most common type with a prevalence of 76% in females followed by 64% in males. Psychosocial factors associated with presence of headache in study subjects were disappointment in relation to academic performance (OR 3.85, CI 1.68-2.71), poor socio-economic status (OR 2.69, CI 1.58-4.57), work overload (OR 0.41, CI 0.24-0.68), irritability (OR 0.33, CI 0.19-0.57) and frequent conflicts (OR 1.45, CI 0.78-2.70). Stress (OR 0.27, CI 0.11-0.71) and anxiety (OR 3.45, CI 1.31-9.08) were associated with headaches only in females and depression (OR 0.50, CI 0.25-1.01) was found to be associated with headaches only in males.

Conclusions

Psychosocial factors from the personal sphere like stress, overwork, and anxiety were highly prevalent amongst students and these factors need to be addressed meticulously in order to mitigate the problem of primary headache disorders amongst medical undergraduates.

## Introduction

Headache is becoming the most disabling and challenging problem related to health worldwide on account of its increasing prevalence and its considerable burden on quality of life of the affected individuals [1]. Headache is ranked among the top 10 disabling conditions by the World Health Organization (WHO). Though the overall disease burden of headache is very extensive worldwide, ironically this disease per se is commonly considered only as a symptom. This eventually affects the life quality and day-to-day activities to a greater extent [2].

The International Classification of Headache Disorders (ICHD-3) classifies headache into primary headache disorders which include cluster, migraine, and tension-type headache (TTH), and secondary headache disorders [2,3]. Primary headache disorders are much more common than secondary headache disorders. Primary headaches constitute approximately 90% of headache cases and only 10% of the remaining headaches are secondary in nature [4]. Despite its high prevalence, less is known about the pathophysiology of primary headaches [4,5]. The tension-type headaches have origins that are of muscular nature and resultant muscular pain tends to be relatively dull achy, poorly localized and radiating [5]. Neurological and brain dysfunctions at times initially present as mere cluster headaches and migraine with eventual involvement of the cranial vessel or the trigeminal nerve [4]. 

Although headache is highly prevalent in the general population, it is also the commonest presenting complaint of students from the medical fraternity, considering their excessive exposure to various psychological stressors throughout the medical school curriculum [4,6]. Psychosocial factors such as stress, sleeping problems, peer pressure, family life, financial difficulties, dissatisfaction with studies, etc., are highly prevalent amongst medical students. It is widely accepted that these psychological factors have a signiﬁcant inﬂuence on headaches [5]. It has been proposed that psychological factors are provoking agents, consequences of returning intense pain and personality traits and that they predispose some people to headaches [1,5].

Various epidemiological surveys have revealed an increasing prevalence of headache in medical students. Migraine and TTH are documented to have a prevalence in the range of 11% to 40% and 46% to 91% respectively in different countries [1,7-10]. Academic performance and quality of life are adversely affected when headache episodes are severe and frequent, thus bringing about the limitation to daily activities and work, as well as significantly influencing students' personal and professional behavior and finally resulting in poor performance in academics too [1,2]. Research in speciﬁc populations, for example in medical students, helps in identifying the possible factors which may influence the occurrence of headache. Although the scenario has been studied extensively at a global level, there is a dearth of literature on the prevalence and patterns of headache particularly in the Indian setup, more so from the present study area. In this context the current study was undertaken in undergraduate medicos of a Government Medical College from the Vidarbha region of India with the following objectives: (1) to estimate the prevalence of headache in the study participants, and (2) to study some psychosocial factors related to it.

## Materials and methods

Design and setting of study

The present study was cross‑sectional in design. It was carried out at Government Medical College located in the Vidarbha region of India from January 2023 to February 2023.

Study population

Medical students who have experienced some form of headache in the last 12 months and had headache attacks during the last 12 months were included in this study. These inclusion criteria were specifically applied with the consideration so as to cover the maximum number of affected students according to the ICHD-3.

Sample

The sample size was calculated based on the prevalence of TTH (79%) obtained by a previous study [11] considering a confidence level of 99% and a margin of error of 5%. Thus the minimum sample size was 442. To meet this required sample size the researchers approached 500 eligible study subjects as per the inclusion criteria. Of these a total of 471 students consented to participate and thus constituted the final sample.

Ethics

Prior to the study, informed written consent was taken from all the participants. Exclusion criteria were as follows: (1) Students who completed less than half of the structured questionnaire; (2) having headache not classified according to the criteria of ICHD-3. The study protocol was approved by the Institutional Ethics Committee.

Data collection

The data was collected by interview techniques. These interviews were conducted in person with the aid of a semi-structured questionnaire by trained senior medical students supervised by a neurologist. The interviewers who collected data from the students were initially briefed by sharing pertinent literature with them which was followed by a formal training from an expert neurologist. This training included conducting interviews on 10 subjects under supervision. This data was excluded from the final analysis.

The questionnaire comprised open and closed type of questions and had a total of three sections. Questions in these sections were based on those used in previous studies. The first section included questions to obtain data on various demographic characteristics like age of the participant, their gender, social and economic status of the family, history of headache in the family, etc. The second section contained questions that provided details of headache so as to identify the type of headache according to the ICHD-3 which is an algorithmic system to define and classify all known headache disorders and is a widely used pre-validated scale. The third and last section comprised questions regarding psychosocial factors related to headache in the students viz. poor socio-economic status, work overload, stress, depression, anxiety, disappointment in relation to academic performance, estranged family relations, disquiet personal life, insomnia, sleep disturbances, irritability, frequent conflicts and not being married. The respondent was asked about the presence or absence of all these factors. Responses were recorded as yes or no and no grading was done.

Statistical analysis

Data entry was carried out in Microsoft Excel (Microsoft, Redmond, WA, USA). It was cleaned and coded. Further analysis was carried out by use of Statistical Package for Social Sciences (SPSS) software, version 24.0 (IBM Corp., Armonk, NY, USA). Odds ratio with 95% confidence interval was calculated. Statistical significance was considered at a p value of less than 0.05.

## Results

In the current study, all 471 respondents were interviewed of which 255 (54%) were female and 216 (46%) were male, the average age being 21.1±1.5 years with a range of 19-25 years. Most of them (66%) stayed away from home. The majority of them (86%) belonged to upper and upper-middle socioeconomic status and 23.6% of the students had a family history of headache. The details of socio-demographic variables are presented in Table 1.

**Table 1:** Socio-demographic variables of study subjects

Variables		n=471	%
Age (M±SD)		21.1±1.5	
Sex	Male	216	46%
	Female	255	54%
Year of study	2^nd^ year	143	30.4%
	3^rd^ year	167	35.5%
	Final year	161	34.2%
Residence	Home	160	34%
	Away from home	311	66%
Family h/o headache	Yes	111	23.6%
	No	360	76.4%
Socio economic status	Upper	183	38.9%
	Upper middle	220	46.7%
	Middle	55	11.7%
	Upper lower	11	2.3%
	Lower	2	0.04%

**Figure 1:**
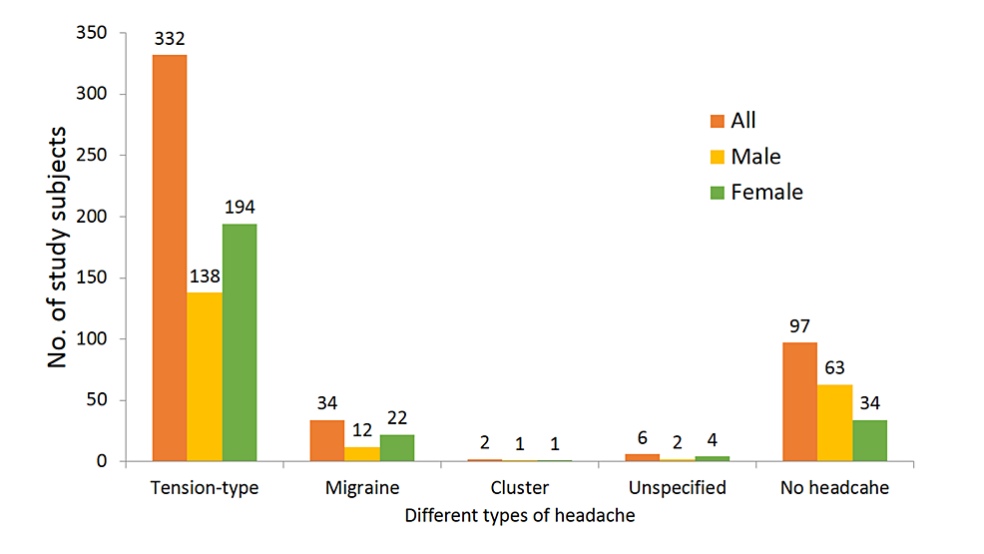
Bar diagram showing the one-year prevalence of different types of headache

The one-year prevalence of different types of headache in male and female students is shown in Figure 1.

Out of 471 students, 374 (80%) students had at least one episode of headache in the previous year. One-year prevalence was higher in females (87%) than males (71%). Tension-type headache is the most prevalent type of headache with its highest prevalence of 76% in females, followed by 71% in all the students together and 64% in males. Migraine was more prevalent in females (9%). However, 97 (21%) of the students did not suffer from headache. 

The presence of different psychosocial factors in the form of poor socio-economic status, work overload, stress, depression, anxiety, disappointment in relation to academic performance, estranged family relations, disquiet personal life, etc., in all the study participants was assessed. The association of these psychosocial factors with the prevalence of headache was analyzed by binary logistic regression. The findings of the same with respect to both the genders collectively considered are depicted in Table 2.

**Table 2:** Binary logistic regression between psychosocial factors and the presence of headache in all the subjects

Sr. No	Psychosocial factors	Headache n=374 (79.5%)	No headache n=97 (20.5%)	Total n=471	OR (95% CI)	P
1.	Disappointment in relation to academic performance	285 (76.2%)	57 (58.7%)	342 (73%)	3.85 (1.68-2.71)	<0.001
2.	Estranged family relations	39 (10.5%)	15 (15.4%)	54 (11.5%)	1.91 (0.95-3.88)	0.06
3.	Disquiet personal life	182 (48.6%)	36 (37%)	218 (46%)	0.62 (0.36-1.05)	0.07
4.	Poor socio-economic status	86 (23%)	36 (37%)	122 (26%)	0.82 (0.48-1.39)	0.46
5.	Stress	147 (39.3%)	31 (32%)	178 (38%)	2.69 (1.58-4.57)	<0.001
6.	Work overload	258 (69%)	47 (48.5%)	305 (65%)	0.41 (0.24-0.68)	<0.001
7.	Sleep disturbances	87 (23.2%)	26 (26.8%)	113 (24%)	1.44 (0.82-2.53)	0.21
8.	Insomnia	112 (30%)	22 (22.6%)	134 (28%)	0.74 (0.42-1.32)	0.31
9.	Depression	146 (39%)	34 (35%)	180 (38%)	1.14 (0.67-1.95)	0.60
10.	Anxiety	186 (49.7%)	37 (38%)	223 (47.3)	0.84 (0.49-1.44)	0.54
11.	Irritability	217 (58%)	33 (34%)	250 (53%)	0.33 (0.19-0.57)	<0.001
12.	Frequent conflicts	85 (22.7%)	21 (21.6%)	106 (22.5%)	1.45 (0.78-2.70)	<0.001

The gender-wise association of all the above-mentioned psychosocial factors with presence or absence of headache for male and female students was also studied separately. The findings of the same are tabulated in Table 3 for male students and in Table 4 for female students.

**Table 3:** Association between psychosocial factors and the presence of headache in female subjects

Sr. No	Psychosocial factors	Headache n=221 (87%)	No headache n=34 (13%)	Total n=255	OR (95% CI)	P
1.	Disappointment in relation to academic performance	187 (84.6%)	24 (70.5%)	211 (83%)	0.45 (0.18-1.12)	0.08
2.	Estranged family relations	18 (8%)	6 (17.6%)	24 (94%)	2.78 (0.89-8.72)	0.07
3.	Disquiet personal life	107 (48.4%)	15 (44.1%)	122 (48%)	1.15 (0.47-2.80)	0.74
4.	Poor socio-economic status	35 (15.8%)	10 (29.4%)	45 (18%)	3.45 (1.31-9.08)	0.01
5.	Stress	74 (33.4%)	7 (20.5%)	81 (32%)	0.53 (0.2-1.45)	<0.001
6.	Work overload	158 (71.4%)	15 (44.1%)	173 (68%)	0.27 (0.11-0.71)	<0.001
7.	Sleep disturbances	41 (18.5%)	8 (23.5%)	49 (19%)	0.45 (0.18-1.25)	0.08
8.	Insomnia	57 (25.7%)	8 (23.5%)	65 (25%)	2.78 (0.89-8.72)	0.07
9.	Depression	86 (38.9%)	10 (29.4%)	96 (38%)	1.15 (0.477-2.80)	0.74
10.	Anxiety	117 (52.9%)	19 (55.8%)	136 (53%)	3.45 (1.31-9.08)	0.01
11.	Irritability	142 (64.2%)	18 (52.9%)	160 (63%)	0.53 (0.20-1.45)	0.22
12.	Frequent conflicts	44 (20%)	8 (23.5%)	52 (20%)	0.27 (0.11-0.71)	0.21

**Table 4:** Association between psychosocial factors and the presence of headache in male subjects

Sr. No	Psychosocial factors	Headache n=153 (71%)	No headache n=63 (29%)	Total n=216 (%)	OR (95% CI)	P
1.	Disappointment in relation to academic performance	99 (64.7%)	33 (52.3%)	132 (61%)	0.71 0.37-1.33)	0.28
2.	Estranged family relations	21 (13.7%)	9 (14.2%)	30 (14%)	1.51 (0.61-3.74)	0.37
3.	Disquiet personal life	75 (49%)	21 (33.3%)	96 (44.5%)	0.50 (0.25-1.01)	0.52
4.	Poor socio-economic status	52 (33.9%)	26 (41.2%)	78 (36%)	1.87 (0.97-3.62)	0.03
5.	Stress	74 (48.3%)	24 (38%)	98 (45%)	0.75 (0.38-1.47)	0.41
6.	Work overload	100 (65.3%)	32 (50.7%)	132 (61%)	0.66 (0.33-1.32)	0.04
7.	Sleep disturbances	46 (30%)	18 (28.5%)	64 (30%)	0.71 (0.73-1.33)	0.28
8.	Insomnia	55 (36%)	14 (22.2%)	69 (32%)	1.51 (0.61-3.74)	0.37
9.	Depression	60 (39.2%)	24 (38%)	84 (39%)	0.50 (0.25-1.01)	0.04
10.	Anxiety	69 (45%)	18 (28.5%)	87 (40%)	1.87 (0.97-3.62)	0.06
11.	Irritability	75 (49%)	15 (23.8%)	90 (42%)	0.75 (0.38-1.47)	<0.001
12.	Frequent conflicts	13 (8.5%)	41 (65%)	54 (25%)	0.66 (0.33-1.32)	0.24

Most of the factors were significantly associated with presence of headache in all students: namely, disappointment in relation to academic performance (OR 3.85, CI 1.68-2.71), poor socio-economic status (OR 2.69, CI 1.58-4.57), work overload (OR 0.41, CI 0.24-0.68), irritability (OR 0.33, CI 0.19-0.57) and frequent conflicts (OR 1.45, CI 0.78-2.70).

When these psychosocial factors were analyzed separately according to gender, a different set of factors were found to be associated with the presence of headache in both males and females. In males, the following factors were found to be associated with headache: bad financial situation (OR 1.87, CI 0.97-3.62), overwork (OR 0.66, CI 0.33-1.32), depressed mood (OR 0.50, CI 0.25-1.01) and irritability (OR 0.75, CI 0.38-1.47). In females, the factors associated with headache were, namely: bad financial situation (OR 3.45, CI 1.31-9.08), overwork (OR 0.53, CI 0.2-1.45), stress (OR 0.27, CI 0.11-0.71) and anxiety (OR 3.45, CI 1.31-9.08).

## Discussion

In the current survey carried out on medical undergraduates to assess headache, it was observed that the gross previous year prevalence of headache was 79.5% which is evidently high. This finding is similar to previous studies conducted by Birru et al. [2] and Elena et al. [12] who reported last year prevalence rate of headache to be 81.1% and 79.6% respectively. However, some studies have shown the last year prevalence of headache to be as low as 54% [4,6]. Hence, there is a wide range of one-year prevalence of headache amongst medical students, from 46% to 91% [7-10]. This discrepancy in prevalence rate could be due to differences in geographical locations social and cultural characteristics and genetic constitutions. The female medical students reported a comparatively higher prevalence of headache (87%) in our study. This finding is similar to findings from previous studies [5,6,11,12]. The higher prevalence of headache in females could be attributed to hormonal influences which eventually affects their psychological responses to various stressful conditions. In this study the prevalence of tension-type headache was also high (71%) in comparison with that of migraine (6.4%). Literature reveals a varying prevalence of migraine ranging from 6.4%-28.7% [1,4,7,13,14]. Tension-type headache had a prevalence ranging from 18.1% to 71.5% as reported by various researchers [1,4,7,10]. Migraine was more prevalent in females (9%) compared to males (5.5%) in our study. It is widely accepted that migraine is more prevalent in females owing to their exposure to wide psychosocial stressors compared to males [2,5,6,12].

Psychosocial factors associated with headache in all students. Stress was indicated by 38% of the students in our study. Students who had stress were 2.7 times more prone to headache than those students with no stress (OR 2.7, CI 1.58-4.57). It is widely accepted that stress is one of the major trigger factors of headache and is highly prevalent amongst medical students ranging from 32% -80% [1,2,5,6,11,12]. Tooba et al. [11] and Elena et al. [12] have shown the association between stress and presence of headache with p values of 0.02 and 0.03 respectively. Medical students, owing to their lack of adequate free time and adapting to a challenging academic environment, might lead to stressful life which leads induction of headache [1,2].

Most of the students (71%) reported that they were dissatisfied with their studies and academic performance. Also, statistically the odds of being dissatisfied with academic performance is 3.8 times more in students suffering from headache (OR 3.8, CI 1.68-4.12). Results from various studies have suggested that academic performance is adversely impacted by headache. Elena et al. [12] stated that odds of having poor academic performance was three times more in students suffering from headache. This being a cross-sectional study, data was collected at a single cross-section of time. Hence it was not possible to establish a direct causal association between headache and students' worse academic performance.

A total of 65% of the students stated that they were overloaded with heavy work. It was statistically signiﬁcantly associated with headache (p=<0.001). A study conducted by other researcher has shown the similar association with p=<0.001 [12]. However, this factor was not analysed before in the majority of the studies.

Irritability (53%) and tendency towards conﬂicts (23%) were also common psychosocial factors associated with headache in all students (p=<0.001) for both. Only a few studies have found the prevalence of these two factors to be 58% and 4.4%-16% respectively [1,12]. One study conducted by Elena et al. [12] proved the association between headache and irritability with a p-value of <0.001. However, the factor of tendency towards conﬂicts was not studied before.

Certain factors played an important role in causing migraine in female undergraduates. We found that some sex-speciﬁc psychosocial factors had an association with headache. Anxiety was not associated with headache when all the students were considered together. It was associated with headache when only female students were considered separately. Similarly, depressed mood was associated with headache in only male students. Bad financial situation was associated with headache in both males and females separately, however no such association was found when all students were considered together.

Fifty-three percent of the female students had suffered from anxiety and the odds of having headache is 3.5 times more in students with anxiety (OR 3.5, CI 1.31-9.08). Several studies have shown the association between these two factors. Anxiety was associated with TTH (p=0.037) and migraine (p=<0.001) in studies conducted by Noor et al. [11] and Elene et al. [12] respectively. It was reported in a community-based study that migraine was commoner in patients with anxiety as compared to healthy controls [15]. Quality of life in migraine patients having anxiety and depression was poorer than that of the control group in another population-based study. It was also observed that the psychiatric comorbidities negatively impacted severity of pain and the number of headache episodes [16].

In males, depressed mood is present in 39% of students and is associated with headache (p=0.04). Self reporting of depression was resorted to in the present study rather than usage of any depression rating scale We did not diagnose depression using rating scales but used self-report. Studies have found its prevalence to be 24% - 32% [11,12]. The commonest psychiatric disorder in headache patients was reported to be depression as per the results of various studies in medical studies [17,18]. However, its association with headache specifically in male students was not established.

Poor socioeconomic status was reported by 18% of females and 36% of males and found to be associated with headache (p=0.01 and p=0.03, respectively). Another study by Panigrahi et al. [1] analyzed socio-economic factors and found that 14% of females and 39% of males reported having a bad financial situation. However, they didn’t show its association with headache.

Some factors like dissatisfaction with family life (94% in females) and dissatisfaction for personal reasons (48% in females and 45% in males) were found to be highly prevalent. Their comparison was not possible as studies that included these factors were limited.

Limitations

The study has a few limitations partly because of its cross-sectional design wherein it is not completely possible to rule out recall bias. Diagnosis of headache was primarily based on self-reporting as per the IHS criteria. Considering the scope of this study, detailed sub-classification of migraine into probable and definite migraine was not done. The study findings may be generalizable to similar study populations and geographical areas.

## Conclusions

Based on the results of this study it can be concluded that the problem of headache is highly prevalent amongst undergraduate students of medical colleges. TTH is the commonest type followed by migraine. Both TTH and migraine were more common in females than in males. Psychosocial factors from the personal sphere like stress, overwork, and anxiety, were highly prevalent amongst students and were signiﬁcantly associated with headache. These factors demand attention so that focused efforts can be directed towards prevention of primary headache in medical students. As stress is a part and parcel of medical education because of the vast syllabus and extreme competitive nature of the profession, measures should be taken to provide a stress-free environment in medical schools. As headache can affect the quality of life of this vulnerable population, attempts should be made at all levels to curb this public health problem.
